# Spontaneous Embedding of DNA Mismatches Within the RNA:DNA Hybrid of CRISPR-Cas9

**DOI:** 10.3389/fmolb.2020.00039

**Published:** 2020-03-17

**Authors:** Brandon P. Mitchell, Rohaine V. Hsu, Marco A. Medrano, Nehemiah T. Zewde, Yogesh B. Narkhede, Giulia Palermo

**Affiliations:** ^1^Department of Bioengineering, University of California, Riverside, Riverside, CA, United States; ^2^Department of Chemistry, University of California, Riverside, Riverside, CA, United States

**Keywords:** CRISPR-Cas9, off-target effects, protein-nucleic acid interactions, molecular dynamics, RNA:DNA hybrid

## Abstract

CRISPR-Cas9 is the forefront technology for editing the genome. In this system, the Cas9 protein is programmed with guide RNAs to process DNA sequences that match the guide RNA forming an RNA:DNA hybrid structure. However, the binding of DNA sequences that do not fully match the guide RNA can limit the applicability of CRISPR-Cas9 for genome editing, resulting in the so-called off-target effects. Here, molecular dynamics is used to probe the effect of DNA base pair mismatches within the RNA:DNA hybrid in CRISPR-Cas9. Molecular simulations revealed that the presence of mismatched pairs in the DNA at distal sites with respect to the Protospacer Adjacent Motif (PAM) recognition sequence induces an extended opening of the RNA:DNA hybrid, leading to novel interactions established by the unwound nucleic acids and the protein counterpart. On the contrary, mismatched pairs upstream of the RNA:DNA hybrid are rapidly incorporated within the heteroduplex, with minor effect on the protein-nucleic acid interactions. As a result, mismatched pairs at PAM distal ends interfere with the activation of the catalytic HNH domain, while mismatches fully embedded in the RNA:DNA do not affect the HNH dynamics and enable its activation to cleave the DNA. These findings provide a mechanistic understanding to the intriguing experimental evidence that PAM distal mismatches hamper a proper function of HNH, explaining also why mismatches within the heteroduplex are much more tolerated. This constitutes a step forward in understanding off-target effects in CRISPR-Cas9, which encourages novel structure-based engineering efforts aimed at preventing the onset of off-target effects.

## GRAPHICAL ABSTRACT

Molecular basis of off-target effects in CRISPR-Cas9.

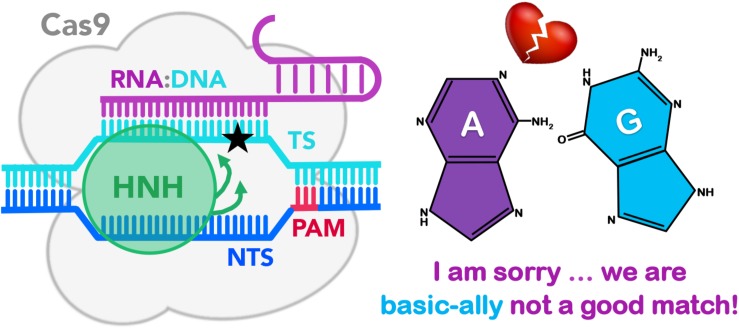

## Introduction

**FIGURE 1:**
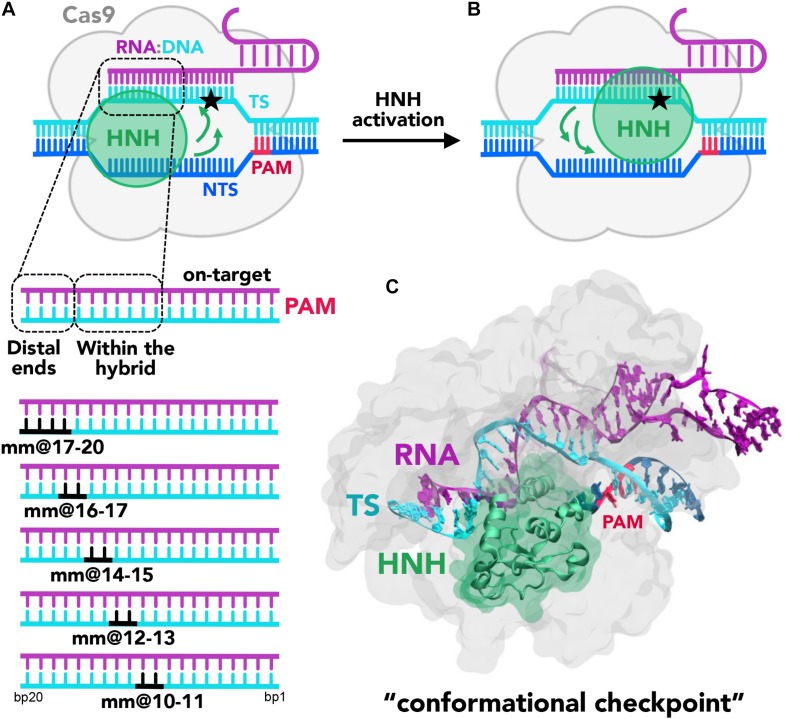
**(A,B)** Cartoon of the endonuclease Cas9 (gray) in complex with the nucleic acids. The DNA target strand (TS, cyan) base pairs the guide RNA (magenta), forming an RNA:DNA hybrid, while the DNA non-target strand (NTS, blue), which also includes the PAM recognition region (red), is displaced. Two conformational states of the catalytic HNH domain (green) are shown. In **(A)**, HNH assumes an inactive “conformational checkpoint” state, which requires a conformational transition (indicated using green arrows) to reach the activated state **(B)**, where it approaches the cleavage site on the TS (indicated using a star). On the bottom panel, a close-up view of the RNA:DNA hybrid highlights the regions at PAM distal ends and within the heteroduplex. In this work, base pair mismatches “mm” have been introduced at positions 17–20, 16–17, 14–15, 12–13, and 10–11 (shown in black). **(C)** X-ray structure of CRISPR-Cas9 identifying the “conformational checkpoint” state (Fu et al., 2013). The protein is shown in molecular surface, with the HNH domain in green. The nucleic acids are shown as ribbons, color-coded as in the cartoon in panel **(A)**.

CRISPR (clustered regularly interspaced short palindromic repeats)-Cas9 is the core of a transformative genome editing technology that is innovating life science with cutting-edge impact in basic and applied biosciences (Doudna and Charpentier, 2014; Hsu et al., 2014). This technology is based on a protein/nucleic acid complex, composed of the endonuclease Cas9, which associates with guide RNAs to recognize and cleave complementary DNA sequences (Figure 1; Jinek et al., 2012). The Cas9 protein performs a site-specific recognition of the DNA, by binding a short sequence of 2–5 nucleotides, known as a Protospacer-Adjacent Motif (PAM), located within the DNA (Sternberg et al., 2014). Upon PAM binding, the DNA base pairs guide the RNA with one strand (i.e., the so-called target strand, TS) to form an 20 base-paired RNA:DNA hybrid structure, while the other DNA non-target strand (NTS) is displaced and subsequently accommodated in the protein.

The formation of a well-matched RNA:DNA hybrid is a fundamental step of the CRISPR-Cas9 function (Sternberg et al., 2015). Indeed, upon formation of the RNA:DNA hybrid, the catalytic HNH domain can change conformation from an inactive state (in which the catalysis is hampered, Figure 1A; Anders et al., 2014; Nishimasu et al., 2014) to a catalytically active conformation, which approaches the cleavage site on the TS (Figure 1B; Jiang et al., 2016). In spite of this fundamental requirement, the presence of DNA mismatches at specific positions of the RNA:DNA hybrid still enables the partial activation of the HNH domain (Fu et al., 2013; Hsu et al., 2013). This leads to the off-target cleavages, which limit the applicability of CRISPR-Cas9, resulting in mutations at sites in the genome other than the desired target site. Several biophysical studies have investigated the effect of base pair mismatches within the RNA:DNA hybrid on the conformational dynamics of CRISPR-Cas9 (Singh et al., 2016; Chen et al., 2017; Dagdas et al., 2017; Yang et al., 2018). Single molecule and kinetics studies have revealed that the presence of 4 base pair mismatches at PAM distal ends can trap the catalytic HNH domain in an inactive conformation also referred to as “conformational checkpoint” (Figure 1, shown as a cartoon in panel A and as a 3D structure in panel B) (Dagdas et al., 2017). As a consequence, the cleavage of the TS gets hampered owing to lack of conformational changes that bring HNH in immediate vicinity to the cleavage site. Inversely, up to 3 base pair mismatches at PAM distal ends still allow the repositioning of HNH, thereby resulting in off-target cleavages. These studies indicate the occurrence of off-target cleavage is linked to the conformational states of HNH. In a recent computational study, we employed molecular dynamics (MD) simulations to investigate the factors affecting the HNH conformational dynamics prior to activation (Ricci et al., 2019). Our study employed the Gaussian accelerated MD (GaMD) method (Miao et al., 2015), to broadly explore the conformational space of CRISPR-Cas9 in complex with an on-target DNA and in the presence of base pair mismatches. These simulations have revealed that the presence of 4 base pair mismatches at PAM distal sites (i.e., at positions 17–20 of the RNA:DNA hybrid) induced an extended opening of the RNA:DNA hybrid, with formation of conserved interactions between the TS and the HNH domain. This effectively decreased the conformational mobility of the HNH domain. Contrariwise, up to 3 base pair mismatches (at positions 18–20) display a lower conformational effect on the RNA:DNA hybrid, and do not affect the conformational dynamics of HNH. These simulations thereby provided a theoretical rationale for the experimental evidence describing the molecular interactions that “lock” HNH in the presence of 4 base pair mismatches at PAM distal ends (Chen et al., 2017; Dagdas et al., 2017; Yang et al., 2018).

However, mechanistic investigations of how DNA mismatches located upstream of the RNA:DNA heteroduplex affect the conformational dynamics of the hybrid structure and the HNH “conformational checkpoint” are absent. Knowledge of the conformational changes arising from base pair mismatches in the middle of the RNA:DNA hybrid are important to gain a deeper understanding of the molecular determinants of off-target binding, which consequently may offer insights for improving the specificity of CRISPR-Cas9. Moreover, understanding how base pair mismatches affect the RNA:DNA structure is important to characterize the dynamics of the heteroduplex itself. This is a key point considering the importance of RNA:DNA hybrids in a variety of biological processes, such as transcription, formation of Okazaki’s fragments and R-loop structures, as well as in eukaryotic chromosomes (Cheatham and Kollman, 1997; Rich, 2006; Shaw and Arya, 2008; Nadel et al., 2015; Palermo, 2019a; Terrazas et al., 2019).

In this research report, we extend our recent investigations to 4 additional model systems, which include base pair mismatches upstream of the RNA:DNA hybrid (Figure 1). Analysis of the results has been performed in comparison with our recently published data, Ricci et al. (2019) thereby evaluating similarities and differences with base pair mismatches at PAM distal ends and with an on-target DNA. We show that while base pair mismatches at PAM distal sites induce an opening of the RNA:DNA hybrid, at upstream positions they are incorporated within the heteroduplex, with minor effect on the protein-nucleic acid interactions. Additionally, mismatches at PAM distal sites limit the mobility of HNH in the “conformational checkpoint” state and consequently affect its activation toward DNA cleavage. Conversely, mismatched pairs within the heteroduplex do not affect the dynamics of HNH, which can freely change conformation as needed to perform DNA cleavages.

## Results and Discussion

To understand the effect of DNA mismatch pairs within the RNA:DNA hybrid on the conformational dynamics of CRISPR-Cas9 and on the HNH domain, we carried out molecular simulations. These investigations have been carried out in analogy to our recent study, which has investigated the effect of mismatch pairs at PAM distal ends (Ricci et al., 2019). In detail, molecular simulations have been performed on the X-ray structure of CRISPR-Cas9 capturing a “conformational checkpoint” state of the HNH domain (i.e., 4UN3.pdb) (Anders et al., 2014), thereby enabling us to understand if and how base pair mismatches could affect the dynamics of HNH prior its activation. A GaMD method has been employed (Miao et al., 2015), adding a boost potential to the simulation that accelerates transitions between low-energy states (see section “Materials and Methods”). The method has been shown to enhance a broad sampling of the conformational space in large biomolecular systems (Miao and McCammon, 2016, 2018; Wang and Chan, 2017; Liao and Wang, 2018; Sibener et al., 2018), including CRISPR-Cas9 as apo form and in complex with nucleic acids (Palermo et al., 2017; Palermo, 2019b), or bound to off-target DNAs (Ricci et al., 2019). Recently, GaMD has shown to sample long time scale motions in agreement with NMR relaxation experiments, showing that the method can efficiently capture the dynamics of large protein/nucleic acid complexes (East et al., 2020). A set of model systems have been built; introducing couples of base pair mismatches “mm” within the hybrid complex at positions 10 to 17 (i.e., mm@10–11, @12–13, @14–15, and @16–17, Figure 1A, bottom panel). The dynamics of these systems have been compared with the simulations of CRISPR-Cas9 binding to an on-target DNA and including 1 to 4 mismatches at PAM distal sites (i.e., mm@17–20, @18–20, @19–20, and @20), which we have recently published (Ricci et al., 2019). For each system, ∼1 μs of conformational sampling has been performed (see section “Materials and Methods”), as in our previous study and by employing the same simulations conditions, thereby enabling proper comparison.

### Dynamics of the RNA:DNA Hybrid in the Presence of DNA Mismatches

**FIGURE 2:**
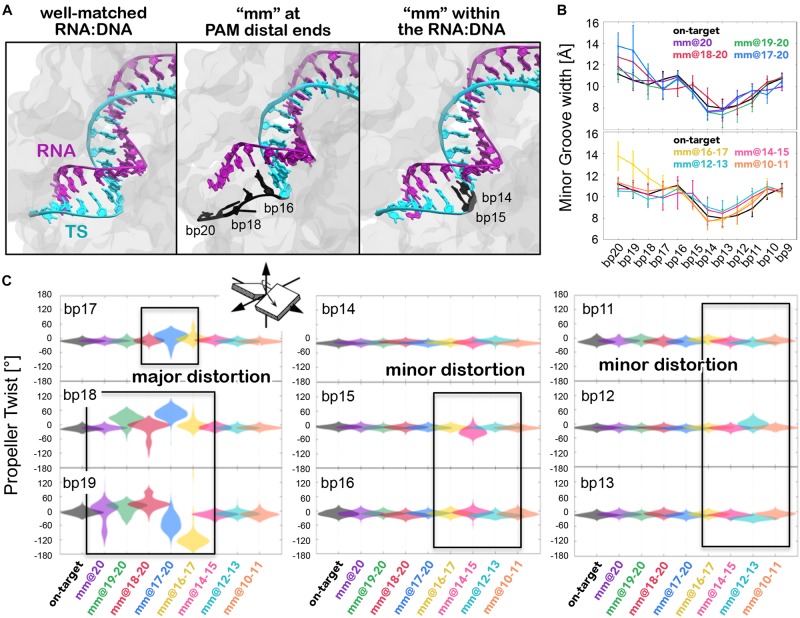
**(A)** Conformations adopted by the RNA:DNA hybrid, in the presence of an on-target DNA (left), including base pair mismatches “mm” at PAM distal ends (center) and within the heteroduplex (right). **(B)** Minor groove width measured at different levels of the RNA:DNA hybrid (i.e., from base pair bp20 to bp9) in the systems including “mm” at PAM distal ends (top panel) and within the heteroduplex (bottom panel). Data are compared with the on-target system. **(C)** Each graph reports the probability distribution (as violin plot) of the *Propeller Twist* angle for each base pair (bp) from PAM distal ends (bp19 to bp17) up to the middle of the RNA:DNA hybrid (bp16 to bp13), computed along the dynamics of each simulated system (reported on the *x*-axis). Regions of major and minor distortions are highlighted using boxes.

Molecular dynamics simulations of CRISPR-Cas9 bound to a fully matched RNA:DNA hybrid (i.e., on-target system) have revealed a stable Watson-Crick base pairing (Figure 2A, left panel), both at PAM distal ends and within the heteroduplex. Notably, transient openings at the end of a DNA duplex, or base flipping are not unusual over long timescales in MD simulations, as shown by several research groups (Pérez et al., 2007, 2008; Mura and McCammon, 2008; Ricci et al., 2010; Ma et al., 2016). However, in the simulations of the on-target CRISPR-Cas9 system, the RNA:DNA hybrid maintains the Watson-Crick base pairing, stabilized by the protein framework, as observed in several conventional and GaMD simulations of this system (Palermo et al., 2016, 2017). Contrariwise, in the presence of base pair mismatches at PAM distal ends (i.e., at positions 16 to 20), we previously observed the opening of the RNA:DNA hybrid (central panel) (Ricci et al., 2019). Here, when we introduce DNA mismatches at the upstream positions (i.e., @10–11, @12–13, and @14–15), we detect that the RNA:DNA hybrid preserves its overall shape (right panel), similarly to what observed in the on-target system. In order to estimate the conformational changes of the RNA:DNA hybrid, we analyzed in all simulated systems, the minor groove width from PAM distal ends up to the middle of the RNA:DNA hybrid (Figure 2B). As a result, we observe that the presence of base pair mismatches at PAM distal ends (i.e., mm@17 to 20) induced an increase of the minor groove width at positions 18–20, which corresponds to the hybrid opening. Notably, the hybrid opening is also observed when including mismatches at positions 16 and 17. This indicates that, perturbations at position 17 (as in the mm@17–20 and mm@16–17 systems) lead to major distortions in the heteroduplex. Conversely, when introducing mismatches at positions 10–11, 12–13, and 14–15, the minor grove width of the RNA:DNA hybrid preserves the conformation of the on-target system.

To understand the effects of the base pair mismatches on the Watson-Crick base pairing, we have used a key geometrical descriptor of the base pair complementarity. We have selected the Propeller Twist parameter (Figure 2C), which describe the rotation of couples of base pairs with respect to each other. Based on our previous study, this parameter enables us to properly characterize alterations in the base pairing along the RNA:DNA hybrid (Ricci et al., 2019). Figure 2C reports the distribution of the Propeller Twist angle along the dynamics for each base pair from PAM distal ends up to the middle of the RNA:DNA hybrid (i.e., from base pair bp20 to bp9). This analysis shows that the presence of base pair mismatches at positions 16 to 20 induces the remarkable loss of base pairing at PAM distal ends, as shown in the mm@20, mm@19–20, mm@18–20, mm@17–20, and in the mm@16–17 systems (“major distortion” in Figure 2C). On the contrary, the geometrical requirements for the base pairing reveal “minor distortion” for mismatches within the RNA:DNA hybrid (i.e., mm@10–11, mm@12–13, and mm@14–15). Notably, this local distortion is due to the loss of base pair interactions (mainly H-bonds), which is typical between DNA mismatched pairs. However, the analysis of the minor grove width (Figure 2B) shows that the hybrid preserves its overall shape when base pair mismatches are introduced in the middle of the structure. Hence, a combined analysis of the minor grove width and the base pair complementarity reveal that the presence of base pair mismatches within the hybrid does not influence the overall shape of the RNA:DNA hybrid, and that base pair mismatches result embedded within the heteroduplex structure.

### Mobility of the HNH Domain in the Presence of DNA Mismatches

**FIGURE 3:**
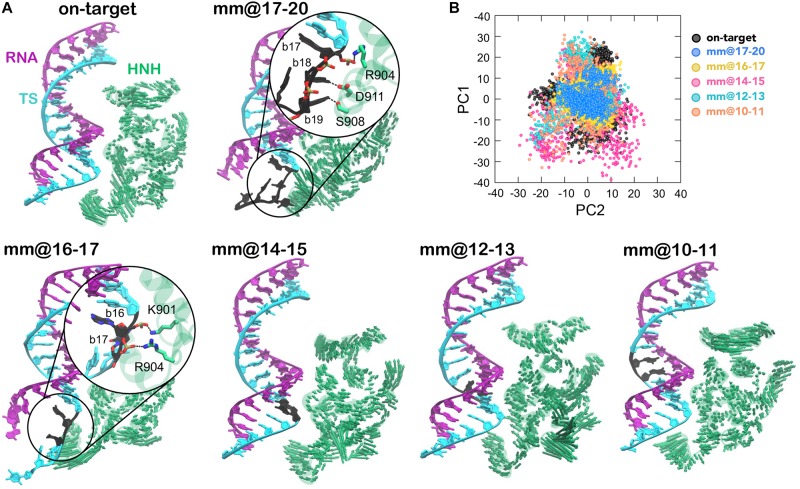
**(A)** “Essential dynamics,” (Amadei et al., 1993) derived from the first principal component (PC1), of the HNH domains in CRISPR-Cas9, binding an on-target DNA and base pair mismatches “mm” at positions 17–20 (top panel), 16–17, 14–15, 12–13, and 10–11 (bottom panel). PC1 is plotted on the three-dimensional structure of HNH (green) using arrows of sizes proportional to the amplitude of motions. The RNA:DNA hybrid is also shown. For the mm@17–20 and mm@16–17 systems, a close-up view shows the interaction between the unwound non-target strand and the HNH domain. **(B)** Projections of the first and second principal motions (PC1 vs. PC2) for the HNH domain in the simulated systems (listed in the legend).

Our previous study has revealed that in the presence of 4 base pair mismatches at PAM distal ends, the DNA TS establishes conserved interactions with the HNH domain (Ricci et al., 2019). These interactions restrict the mobility of HNH and affect its conformational activation toward DNA cleavage, while also contributing to the widening of the RNA:DNA hybrid. Here, in order to assess the conformational mobility of HNH in the presence of base pair mismatches within the RNA:DNA hybrid, we performed Principal Component Analysis (PCA). This analysis enabled to capture the essential degrees of freedom of the HNH domain (see section “Materials and Methods”). PCA has been carried out in comparison with the on-target system and with the system including 4 base pair mismatches at PAM distal ends (i.e., mm@17–20). Figure 3A reports the dynamics of the HNH domain along its first mode of motion (i.e., Principal Component 1, PC1), shown using arrows to indicate the direction and relative amplitude of the motions. The top panel shows a comparison between the system binding an on-target DNA and in the presence of 4 base pair mismatches at positions 17–20. In the mm@17–20 system, we observe that the unwound TS approaches the arrows corresponding to the HNH principal motion. A close-up view displays the interactions established by the DNA and the residues of the HNH domain. Notably, these interactions are stable along the dynamics, as discussed in our previous paper. The bottom panel reports the PCA analysis for the simulated systems including base pair mismatches within the RNA:DNA hybrid. We observe that for base pair mismatches at positions 16–17, the TS displays a similar unwinding of the mm@17–20 system, with conserved interactions established with the HNH domain (close-up view). Indeed, the interaction between the nucleobases at position 17 and R904 is conserved in the two systems. This indicates that local distortions due to mismatched nucleobases at position 17, which is in close proximity to the HNH (α-helices, can critically affect the dynamics of HNH. We note that the interaction established at position 17 involves the DNA backbone (rather than the nucleobases), which suggests that this interaction is not specific, but rather could be established also in the presence of different mismatched nucleobases. This hypothesis, however, warrants further investigations, which are currently ongoing in our lab as a follow-up of this study. On the contrary, base pair mismatches @10–11, @12–13, and @14–15 do not result in the approach of the TS to the HNH domain, resembling what observed the dynamics of the on-target system (top panel).

In order to characterize the conformational space sampled by the HNH domain, we plotted the first versus the second principal components (PC1 vs. PC2, Figure 3B). This analysis revealed that in the mm@17–20 system, HNH explores a narrower conformational space with respect to the remaining systems, indicating a diminished mobility. A narrow conformational space is also observed for the mm@16–17 system. As discussed above, in these two systems, the TS tightly interacts with the HNH domain, thereby limiting its conformational dynamics. In the systems including base pair mismatches within the RNA:DNA hybrid, the HNH domain assumes a wider conformational space, similar to what observed in the on-target system. This indicates that the dynamics of HNH is not significantly affected by base pair mismatches in the middle of the RNA:DNA hybrid.

**FIGURE 4:**
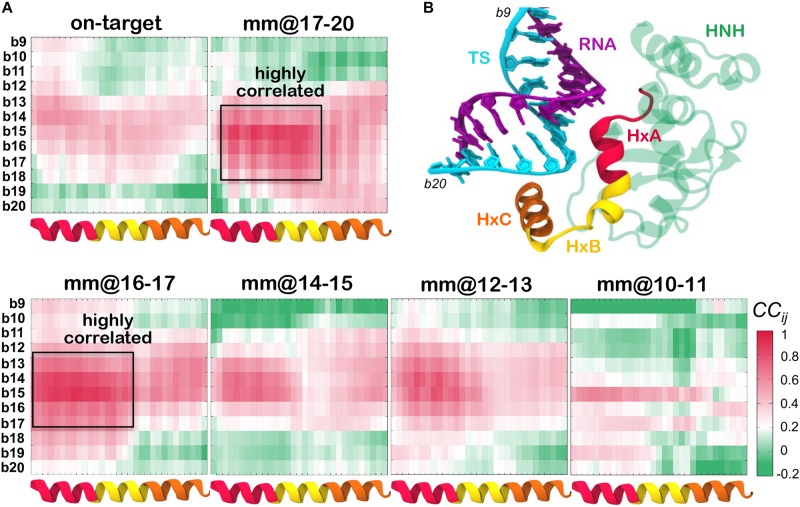
**(A)** Cross-Correlation (*CC*_*ij*_) matrices computed between the HNH α-helices that locate in proximity of the hybrid (*x*-axis), and the DNA TS from position b20 to position b9 (*y*-axis). The *CC*_*ij*_ coefficients are computed between the protein Cα and the DNA phosphate atoms. Data are reported for CRISPR-Cas9 binding an on-target DNA and including base pair mismatches “mm” at positions 17–20 (top panel), as well as with “mm” at positions 16–17, 14–15, 12–13, and 10–11 (bottom panel). Positive correlations (*CC*_*ij*_ ≥ 0) are shown in magenta, whereas anti-correlated motions display negative correlations (*CC*_*ij*_ ≤ 0) are shown in green (legend on the bottom right). Two boxes indicate highly coupled motions in the mm@17–20 and mm@16–17 systems. **(B)** Cartoon of the system, displaying the regions used to compute the *CC*_*ij*_ matrix. The HNH domain is shown as cartoon (green), with the α-helices HxA (residues 890–900, red), HxB (residues 901–910, yellow) and HxC (residues 911–920, orange) in different colors. The RNA (violet) and the DNA TS (cyan) are shown as ribbons.

To further characterize the mobility of the systems and to understand the relation between the dynamics of the nucleic acids and the HNH domain, we performed cross-correlation (*CC*_*ij*_) analysis. This analysis enabled us capturing coupled motions between the protein Cα atoms and the TS phosphate atoms (details in the see section “Materials and Methods”). Figure 4A reports the *CC*_*ij*_ matrices computed between the residues of the HNH α-helices that locate in proximity of the hybrid, and the TS bases from position b20 (PAM distal ends) to position b9 (within the hybrid). Positive correlations (*CC*_*ij*_ = 0, magenta) indicate highly coupled motions in the same direction, whereas anti-correlated motions display negative correlations (*CC*_*ij*_ = 0, green). A cartoon of the system, highlighting the regions used to compute the cross-correlations is shown in Figure 4B. For the sake of the clarity, the HNH α-helices in proximity of the hybrid are indicated in red (residues 890–900, Helix–A), yellow (901–910, Helix–B) and orange (911–920, Helix–C).

As a result of this analysis, in the presence of mismatches at PAM distal ends (i.e., in the mm@17–20 system) and at positions 16–17 (mm@16–17 system), Helix–A and Helix–B are highly correlated with the TS bases from position 18 to 14 (as highlighted using a box in Figure 4A). This indicates that the dynamics of the HNH and of the TS are mutually affected by each other, when in the presence of mismatched pairs at PAM distal ends. Moreover, we note that in the presence of mismatches at PAM distal ends, the DNA TS mainly interacts with Helix–B (Figure 3A, and also shown by Ricci and coauthors) (Ricci et al., 2019), thereby affecting its conformational dynamics. Inversely, in the systems displaying base pair mismatches at upstream positions (mm@14 to 10), as well as in the on-target system, a weakening of the correlated motions can be seen. In these systems, there are no interactions being established between the TS and the HNH domain, signified by the diminished correlations between them. Overall, the cross-correlation analyses confirm that the presence of base pair mismatches at PAM distal ends affects the dynamics of HNH, while mismatches at upstream positions do not exert a relevant effect.

## Conclusion

Here, molecular simulations have been used to characterize the conformational dynamics of CRISPR-Cas9 in the presence of base pair mismatches within the RNA:DNA hybrid. The simulations have shown that the presence of base pair mismatches at PAM distal ends of the RNA:DNA hybrid (i.e., positions 20 to 17) induce an opening of the heteroduplex (Ricci et al., 2019). As a result, newly formed interactions between the DNA TS and the catalytic HNH domain have been shown to “trap” HNH in an inactive “conformational checkpoint” state, hampering its activation for cleavage. On the contrary, base pair mismatches at upstream positions (i.e., within the RNA:DNA hybrid, at positions 14 to 10) are incorporated within the heteroduplex, with minor effect on the protein-nucleic acid interactions. Indeed, the presence of DNA mismatches within the hybrid does not affect the mobility of HNH, which is similar to that of the on-target system (Figure 3). This suggests that mismatched base pairs within the RNA:DNA hybrid do not interfere with the process of HNH activation (Figure 1A), where HNH changes in configuration from its “conformational checkpoint” state to an activated form are prone to cleave the DNA TS (Figures 1A,B). Notably, these results agree with existing experimental studies and offer a rationale to the observed outcomes. Indeed, the presence of DNA mismatches at PAM distal ends has been experimentally shown to trap HNH in a “conformational checkpoint” state, likely due to interactions established with the DNA TS, as previously suggested (Singh et al., 2016; Chen et al., 2017; Dagdas et al., 2017; Yang et al., 2018). However, mismatches in the middle of the hybrid are much more tolerated than at PAM distal ends, and lead to DNA cleavages. In light of this fact, our results indicate that mismatches at upstream positions (i.e., positions 14 to 10) still allow to preserve the overall structure of the RNA:DNA, without affecting the conformational dynamics of the catalytic HNH domain. As such, HNH can freely change conformation as needed to perform DNA cleavages (Figures 1A,B). Overall, this research report constitutes a step forward in understanding the effect of DNA mismatches within the RNA:DNA hybrid in CRISPR-Cas9, offering insightful information on off-target effects. This work also forms the basis for further investigation, to characterize the effect of DNA mismatches along the entire RNA:DNA hybrid and therefore to report an atomic-level understanding also for DNA mismatches at PAM-proximal sites (i.e., positions 1 to 9). These studies are currently ongoing in our laboratory, as inspired from the current work, taking also into account different conformations of the HNH (Figure 1A) domain and diverse mismatched nucleobases. Finally, we note that understanding how mismatched pairs affect the heteroduplex structure is *per se* important to understand the function of RNA:DNAs, which are critical in a variety of biological processes (Cheatham and Kollman, 1997; Rich, 2006; Shaw and Arya, 2008; Nadel et al., 2015; Palermo, 2019a; Terrazas et al., 2019).

In summary, this study provides an atomic-level understanding of the dynamic effects of the binding of DNA base pair mismatches within the RNA:DNA hybrid in CRISPR-Cas9. As a take-home message, the presence of mismatched pairs at distinctive locations of the RNA:DNA hybrid produces different conformational effects, which affect the protein counterpart. Specifically, mismatched pairs at PAM distal ends interfere with the activation of the catalytic HNH domain, while mismatches fully embedded in the RNA:DNA do not affect the HNH dynamics and enable its activation to cleave the DNA. This provides a reasonable explanation on why off-target sequences holding mismatches at PAM distal ends are less likely to produce DNA cleavages in CRISPR-Cas9, than mismatched pairs within the heteroduplex, as experimentally observed (Singh et al., 2016; Chen et al., 2017; Dagdas et al., 2017; Yang et al., 2018). These findings contribute in understanding the mechanistic basis of off-target effects in CRISPR-Cas9 and encourage novel experimental studies aimed at designing more specific variants of the system that prevent the onset of off-target effects.

## Materials and Methods

Structural models have been based on the X-ray structure of the *Streptococcus pyogenes* CRISPR-Cas9 complex (4UN3.pd, 2.58 Å resolution) (Anders et al., 2014) which captures the inactivated state of the HNH domain (i.e., “conformational checkpoint”) (Dagdas et al., 2017). MD simulations have been performed applying a well-established protocol for protein/nucleic acid complexes, which employs the Amber ff12SB force field, including the ff99bsc0 (Perez et al., 2007) corrections for DNA and the ff99bsc0+(χOL3 (Banas et al., 2010; Zgarbova et al., 2011) corrections for RNA. To broadly explore the conformational space of CRISPR-Cas9, we employed a recent accelerated MD (aMD) simulations method (Miao et al., 2015). Specifically, we applied a Gaussian aMD (GaMD) method, which adds a harmonic boost potential to smoothen the potential energy surface, thereby decreasing energy barriers and accelerating transitions between the low-energy states (a complete description of the method is reported as a Supplementary Material). The method has extended the use of aMD to large biomolecular systems, with applications of this method to G-protein coupled receptors (Miao and McCammon, 2016, 2018), the Mu opioid receptor (Wang and Chan, 2017; Liao and Wang, 2018), T-cell receptors (Sibener et al., 2018), and CRISPR-Cas9 (Palermo et al., 2017; Palermo, 2019b; Ricci et al., 2019).

## Data Availability Statement

The datasets generated for this study are available on request to the corresponding author.

## Author Contributions

BM and RH contributed equally. BM and RH performed the simulations, analyzed the data, and wrote the manuscript. MM, NZ, and YN analyzed the data and wrote the manuscript. GP conceived the original research, supervised research, and wrote the manuscript.

## Conflict of Interest

The authors declare that the research was conducted in the absence of any commercial or financial relationships that could be construed as a potential conflict of interest. The reviewer YM declared a past collaboration with one of the authors GP.

## References

[B1] Amadei A. Linssen A. B. M. Berendsen H. J. C. (1993). Essential dynamics of proteins. *Proteins Struct. Funct. Genet.* 17 412–425. 10.1002/prot.340170408 8108382

[B2] Anders C. Niewoehner O. Duerst A. Jinek M. (2014). Structural basis of PAM-dependent target DNA recognition by the Cas9 endonuclease. *Nature* 513 569–573. 10.1038/nature13579 25079318 PMC4176945

[B3] Banas P. Hollas D. Zgarbova M. Jurecka P. Orozco M. Cheatham T. E. III (2010). Performance of molecular mechanics force fields for RNA simulations: stability of UUCG and GNRA hairpins. *J. Chem. Theor. Comput.* 6 3836–3849. 10.1021/ct100481h PMC8916691 35283696

[B4] Cheatham T. E. III Kollman P. A. (1997). Molecular dynamics simulations highlight the structural differences among DNA:DNA, RNA:RNA, and DNA:RNA hybrid duplexes. *J. Am. Chem. Soc.* 11 14805–14825.

[B5] Chen J. S. Dagdas Y. S. Kleinstiver B. P. Welch M. M. Sousa A. A. Harrington L. B. (2017). Enhanced proofreading governs CRISPR–Cas9 targeting accuracy. *Nature* 550 407–410. 10.1038/nature24268 28931002 PMC5918688

[B6] Dagdas Y. S. Chen J. S. Sternberg S. H. Doudna J. A. (2017). A Conformational checkpoint between DNA binding and cleavage by CRISPR-Cas9. *Sci. Adv.* 3:eaao002. 10.1126/sciadv.aao0027 PMC5547770 28808686

[B7] Doudna J. A. Charpentier E. (2014). Genome Editing. The new frontier of genome engineering with CRISPR-Cas9. *Science* 346:1258096. 10.1126/science.1258096 25430774

[B8] East K. W. Newton J. C. Morzan U. N. Narkhede Y. B. Acharya A. Skeens E. (2020). Allosteric motions of the CRISPR–Cas9 HNH nuclease probed by NMR and molecular dynamics. *J. Am. Chem. Soc.* 142 1348–1358. 10.1021/jacs.9b10521 31885264 PMC7497131

[B9] Fu Y. Foden J. A. Khayter C. Maeder M. L. Reyon D. Joung J. K. (2013). High-frequency off-target mutagenesis induced by CRISPR-Cas nucleases in human cells. *Nat. Biotechnol.* 31 822–826. 10.1038/nbt.2623 23792628 PMC3773023

[B10] Hsu P. D. Lander E. S. Zhang F. (2014). Development and aplications of CRISPR-Cas9 for genome engineering. *Cell* 1576 1262–1278. 10.1016/j.cell.2014.05.010 PMC4343198 24906146

[B11] Hsu P. D. Scott D. A. Weinstein J. A. Ran F. A. Konermann S. Agarwala V. (2013). DNA targeting specificity of RNA-Guided Cas9 nucleases. *Nat. Biotechnol.* 31 827–832. 10.1038/nbt.2647 23873081 PMC3969858

[B12] Jiang F. Taylor D. W. Chen J. S. Kornfeld J. E. Zhou K. Thompson A. J. (2016). Structures of a CRISPR-Cas9 R-Loop complex primed for DNA cleavage. *Science* 351 867–871. 10.1126/science.aad8282 26841432 PMC5111852

[B13] Jinek M. Chylinski K. Fonfara I. Hauer M. Doudna J. A. Charpentier E. (2012). A Programmable Dual-RNA-Guided DNA endonuclease in adaptive bacterial immunity. *Science* 337 816–821. 10.1126/science.1225829 22745249 PMC6286148

[B14] Liao J. M. Wang Y. T. (2018). In silico studies of conformational dynamics of mu opioid receptor performed using gaussian accelerated molecular dynamics. *J. Biomol. Struct. Dyn.* 37 166–177. 10.1080/07391102.2017.1422025 29277141

[B15] Ma Z. Palermo G. Adhireksan Z. Murray B. S. von Erlach T. Dyson P. J. (2016). An organometallic compound displays a unique one-stranded intercalation mode that is DNA topology-dependent. *Angew Chem. Int. Ed.* 128 7441–7444. 10.1002/anie.201602145 27184539

[B16] Miao Y. Feher V. A. McCammon J. A. (2015). Gaussian accelerated molecular dynamics: unconstrained enhanced sampling and free energy calculation. *J. Chem. Theor. Comput.* 11 3584–3595. 10.1021/acs.jctc.5b00436 PMC4535365 26300708

[B17] Miao Y. McCammon J. A. (2016). Graded activation and free energy landscapes of a muscarinic G protein-coupled receptor. *Proc. Natl. Acad. Sci. U.S.A.* 113 12162–12167. 10.1073/pnas.1614538113 27791003 PMC5087018

[B18] Miao Y. McCammon J. A. (2018). Mechanism of the G-protein mimetic nanobody binding to a muscarinic G-protein-coupled receptor. *Proc. Natl. Acad. Sci. U.S.A.* 115 3036–3041. 10.1073/pnas.1800756115 29507218 PMC5866610

[B19] Mura C. McCammon J. A. (2008). Molecular dynamics of a KB DNA element: base flipping via cross-strand intercalative stacking in a microsecond-scale simulation. *Nucleic Acids Res.* 36 4941–4955. 10.1093/nar/gkn473 18653524 PMC2528173

[B20] Nadel J. Athanasiadou R. Lemetre C. Wijetunga N. A. O’Broin P. Sato H. (2015). RNA:DNA Hybrids in the human genome have distinctive nucleotide characteristics, chromatin composition, and transcriptional relationships. *Epigenetics Chromatin* 8:46. 10.1186/s13072-015-0040-6 26579211 PMC4647656

[B21] Nishimasu H. Ran F. A. Hsu P. D. Konermann S. Shehata S. I. Dohmae N. (2014). Crystal structure of Cas9 in complex with guide RNA and target DNA. *Cell* 156 935–949. 10.1016/j.cell.2014.02.001 24529477 PMC4139937

[B22] Palermo G. (2019a). Dissecting structure and function of DNA⋅RNA hybrids. *Chemistry* 5 1364–1366. 10.1016/j.chempr.2019.05.015

[B23] Palermo G. (2019b). Structure and dynamics of the CRISPR–Cas9 catalytic complex. *J. Chem. Inf. Model.* 59 2394–2406. 10.1021/acs.jcim.8b00988 30763088

[B24] Palermo G. Miao Y. Walker R. C. Jinek M. McCammon J. A. (2016). Striking plasticity of CRISPR-Cas9 and key role of non-target DNA, as revealed by molecular simulations. *ACS Cent. Sci.* 2 756–763. 10.1021/acscentsci.6b00218 27800559 PMC5084073

[B25] Palermo G. Miao Y. Walker R. C. Jinek M. McCammon J. A. (2017). CRISPR-Cas9 conformational activation as elucidated from enhanced molecular simulations. *Proc. Natl. Acad. Sci. U.S.A.* 114 7260–7265. 10.1073/pnas.1707645114 28652374 PMC5514767

[B26] Pérez A. Lankas F. Luque F. J. Orozco M. (2008). Towards a Molecular Dynamics Consensus View of B-DNA Flexibility. *Nucleic Acids Res.* 36 2379–2394. 10.1093/nar/gkn082 18299282 PMC2367714

[B27] Pérez A. Luque F. J. Orozco M. (2007). Dynamics of B-DNA on the microsecond time scale. *J. Am. Chem. Soc.* 129 14739–14745. 10.1021/ja0753546 17985896

[B28] Perez A. Marchan I. Svozil D. Sponer J. Cheatham T. E. III Laughton C. A. (2007). Refinement of the AMBER force field for nucleic acids: improving the description of Alpha/Gamma conformers. *Biophys. J.* 92 3817–3829. 10.1529/biophysj.106.097782 17351000 PMC1868997

[B29] Ricci C. G. Chen J. S. Miao Y. Jinek M. Doudna J. A. McCammon J. A. (2019). Deciphering off-target effects in CRISPR-Cas9 through accelerated molecular dynamics. *ACS Cent. Sci.* 5 651–662. 10.1021/acscentsci.9b00020 31041385 PMC6487449

[B30] Ricci C. G. de Andrade A. S. C. Mottin M. Netz P. A. (2010). Molecular dynamics of DNA: comparison of force fields and terminal nucleotide definitions. *J. Phys. Chem. B* 114 9882–9893. 10.1021/jp1035663 20614923

[B31] Rich A. (2006). Discovery of the hybrid helix and the first DNA-RNA hybridization. *J. Biol. Chem.* 281 7693–7696. 10.1074/jbc.x600003200 16547011

[B32] Shaw N. N. Arya D. P. (2008). Recognition of the unique structure of DNA:RNA Hybrids. *Biochimie* 90 1026–1039. 10.1016/j.biochi.2008.04.011 18486626

[B33] Sibener L. V. Fernandes R. A. Kolawole E. M. Carbone C. B. Liu F. McAffee D. (2018). Isolation of a structural mechanism for uncoupling t cell receptor signaling from Peptide-MHC binding. *Cell* 174 672–687. 10.1016/j.cell.2018.06.017 30053426 PMC6140336

[B34] Singh D. Sternberg S. H. Fei J. Doudna J. A. Ha T. (2016). Real-time observation of DNA recognition and rejection by the RNA-Guided endonuclease Cas9. *Nat. Commun.* 7:12778. 10.1038/ncomms12778 27624851 PMC5027287

[B35] Sternberg S. H. LaFrance B. Kaplan M. Doudna J. A. (2015). Conformational control of DNA target cleavage by CRISPR-Cas9. *Nature* 527 110–113. 10.1038/nature15544 26524520 PMC4859810

[B36] Sternberg S. H. Redding S. Jinek M. Greene E. C. Doudna J. A. (2014). DNA interrogation by the CRISPR RNA-guided endonuclease Cas9. *Nature* 507 62–67. 10.1038/nature13011 24476820 PMC4106473

[B37] Terrazas M. Genna V. Portella G. Villegas N. Sánchez D. Arnan C. (2019). The origins and the biological consequences of the Pur/Pyr DNA⋅RNA asymmetry. *Chemistry* 5 1619–1631. 10.1016/j.chempr.2019.04.002

[B38] Wang Y.-T. Chan Y.-H. (2017). Understanding the molecular basis of agonist/antagonist mechanism of human Mu Opioid receptor through gaussian accelerated molecular dynamics method. *Sci. Rep.* 7:7828. 10.1038/s41598-017-08224-2 28798303 PMC5552784

[B39] Yang M. Peng S. Sun R. Lin J. Wang N. Chen C. (2018). The conformational dynamics of Cas9 governing DNA cleavage are revealed by single-molecule FRET. *Cell Rep.* 22 372–382. 10.1016/j.celrep.2017.12.048 29320734

[B40] Zgarbova M. Otyepka M. Sponer J. Mladek A. Banas P. Cheatham T. E. (2011). Refinement of the Cornell et al. nucleic acids force field based on reference quantum chemical calculations of glycosidic torsion profiles. *J. Chem. Theory Comput.* 7 2886–2902. 10.1021/ct200162x 21921995 PMC3171997

