# Supplementary material for: Spontaneous Embedding of DNA Mismatches Within the RNA:DNA Hybrid of CRISPR-Cas9
Source: Front Mol Biosci. 2020 Mar 17;7:39. doi: 10.3389/fmolb.2020.00039 (PMC7093078; doi:10.3389/fmolb.2020.00039)
Supplement: Supplementary file 1 [file Data_Sheet_1.pdf]

Supporting Information

Spontaneous embedding of DNA mismatches within the RNA:DNA hybrid of CRISPR-Cas9

Brandon P. Mitchell,[1†] Rohaine V. Hsu,[1†] Marco A. Medrano,[1] Nehemiah T. Zewde,[1] Yogesh B. Narkhede[1] and Giulia Palermo[1,2*]

1. Department of Bioengineering, University of California Riverside, Riverside CA 92507, United States
2. Department of Chemistry, University of California Riverside, Riverside, CA 92507, United States

Corresponding author:
Dr. Giulia Palermo (giulia.palermo@ucr.edu)
[†]These authors contributed equally

Supplementary Materials and Methods

Structural models. Molecular Dynamics (MD) simulations have been based on the X-ray structure of the *Streptococcus pyogenes* (*Sp*) CRISPR-Cas9 in complex with RNA and DNA (4UN3.pdb), solved at 2.58 Å resolution.[1] This structure identifies the inactivated state of the HNH domain (i.e., the "conformational checkpoint").[2] Four model systems have been built, including base pair mismatches "mm" within the RNA:DNA at different positions (i.e., mm@16-17, mm@14-15, mm@12-13, mm@10-11, as in Figure 1A). These structural models have been embedded in explicit waters, while Na+ ions were added to neutralize the total charge, leading to an orthorhombic periodic simulation cell of ~145 · 110 · 145 $Å^3$, containing a total of ~220,000 atoms. Notably, the simulation systems have been built similarly to our recent paper,[3] which investigated the dynamics of CRISPR-Cas9 in the presence of base pair mismatches at positions 17-20, 18-20, 19-20 and 20. The outcomes of these previously published systems have been compared with the results presented in this paper.

Molecular Dynamics (MD). MD simulations have been performed in analogy to our previous paper. In detail, conventional MD simulations have been carried out to equilibrate the systems, prior to Gaussian accelerated MD (GaMD) simulations.[4] MD simulations have been performed using a simulation protocol successfully employed for CRISPR-Cas9[3,5–8] and widely adopted for other RNA/DNA nucelases,[9] using of the Amber ff12SB force field, which includes the ff99bsc0[10] corrections for DNA and the ff99bsc0+χ*OL3*[11,12] corrections for RNA. The Åqvist force field[13] has been employed for Mg^{2+} ions. An integration time step of 2 fs has been employed. All bond lengths involving hydrogen atoms were constrained using the SHAKE algorithm. Temperature control (300 K) has been performed via Langevin dynamics,[14] with a collision frequency γ = 1/ps. Pressure control was accomplished by coupling the system to a

Berendsen barostat,[15] at a reference pressure of 1 atm and with a relaxation time of 2 ps. The system has been subjected to energy minimization to relax water molecules and counter ions, keeping the protein, the RNA, DNA and Mg ions fixed with harmonic position restraints of 300 kcal/mol · $Å^2$. Then, the system has been heated up from 0 to 100 K in the canonical ensemble (NVT), by running two simulations of 5 ps each, imposing position restraints of 100 kcal/mol · $Å^2$ on the above-mentioned elements of the system. The temperature was further increased up to 200 K in ~100 ps of MD in the isothermal-isobaric ensemble (NPT), reducing the restraint to 25 kcal/mol $Å^2$. Subsequently, all restraints were released and the temperature of the system was raised up to 300 K in a single NPT simulation of 500 ps. After ~ 1.1 ns of equilibration, ~ 10 ns of NPT runs were carried out allowing the density of the system to stabilize around 1.01 g/cm^{-3}. Finally, the ~100 ns have been carried out in NVT ensemble. Simulations have been performed using the GPU version of AMBER pmemd 18.[16]

Gaussian accelerated Molecular Dynamics (GaMD). Accelerated MD (aMD) is an enhanced sampling method that works by adding a non-negative boost potential to smoothen the system potential energy surface (PES), thus effectively decreasing the energy barriers and accelerating transitions between the low-energy states.[17] The method has been extensively employed to accelerate protein dynamics in a variety of biomolecules (see Markwick & McCammon as a review).[18] However, the use of aMD for large biomolecular systems, such as CRISPR-Cas9, can suffer from high statistical noise, which hampers the characterization of the correct statistical ensemble.[19–22] To overcome this limitation, we employed here a novel and more robust Gaussian aMD (or Gaussian aMD)[4] method, in which the boost potential follows Gaussian distribution. This allows smoothly reconstructing the original shape of the potential energy surface, through accurate reweighting using cumulant expansion to the 2nd order. This has

expanded the use of aMD to large biological systems, with applications of this method to G-protein coupled receptors,[23,24] the Mu opioid receptor,[25,26] T-cell receptors[27] and CRISPR-Cas9.[3,6,8]

Considering a system with N atoms at positions $\vec{r} = \{\vec{r_1}, \ldots \vec{r_N}\}$, when the system potential $V(\vec{r})$ is lower than a threshold energy E, the energy surface is modified by adding a boost potential as:

$$V^*(\vec{r}) = V(\vec{r}) + \Delta V(\vec{r}), \qquad V(\vec{r}) < E, \tag{1}$$

$$\Delta V(\vec{r}) = \frac{1}{2}k\left(E - V(\vec{r})\right)^2, \tag{2}$$

where k is the harmonic force constant. The two adjustable parameters E and k are automatically determined by applying the following three criteria. First, for any two arbitrary potential values $V_1(\vec{r})$ and $V_2(\vec{r})$ found on the original energy surface, if $V_1(\vec{r}) < V_2(\vec{r})$, ΔV should be a monotonic function that does not change the relative order of the biased potential values, i.e., $V_1^*(\vec{r}) < V_2^*(\vec{r})$. Secondly, if $V_1(\vec{r}) < V_2(\vec{r})$, the potential difference observed on the smoothened energy surface should be smaller than that of the original, i.e., $V_2^*(\vec{r}) - V_1^*(\vec{r}) < V_2(\vec{r}) - V_1(\vec{r})$. By combining the first two criteria and plugging in the formula of $V^*(\vec{r})$ and ΔV, we obtain:

$$V_{max} \leq E \leq V_{min} + \frac{1}{k}, \tag{3}$$

where V_{min} and V_{max} are the system minimum and maximum potential energies. To ensure that Eqn. [4] is valid, k has to satisfy $k \leq \frac{1}{V_{max} -} V_{min}$. By defining $k \equiv k_0 \frac{1}{V_{max} -} V_{min}$, then $0 < k \leq 1$. Thirdly, the standard deviation of ΔV needs to be small enough (i.e., narrow distribution) to ensure accurate reweighting using cumulant expansion to the second order: $\sigma_{\Delta V} =$

$k(E - V_{avg})\sigma_V \leq \sigma_0$, where V_{avg} and σ_V are the average and standard deviation of the system potential energies, $\sigma_{\Delta V}$ is the standard deviation of ΔV and σ_0 as a user-specified upper limit (e.g., 10 kBT) for accurate reweighting. When E is set to the lower bound, $E = V_{max}$, according to Eqn. [4], k_0 can be calculated as:

$$k_0 = (1.0, k_0') = \left(1.0, \frac{\sigma_0}{\sigma_V} \cdot \frac{V_{max}-V_{min}}{V_{max}-V_{avg}}\right). \quad [4]$$

Alternatively, when the threshold energy E is set to its upper bound $E = V_{min} + \frac{1}{k}$, k_0 is:

$$k_0 = k_0'' \left(1 - \frac{\sigma_0}{\sigma_V}\right) \cdot \frac{V_{max}-V_{min}}{V_{avg}-V_{min}}, \quad [5]$$

if k_0'' is calculated between 0 and 1. Otherwise, k_0 is calculated using Eqn. [4], instead of being set to 1 directly as described in the original paper. In Gaussian aMD, even with biasing potential, the same low-energy physical states are sampled, such enabling quantitative recovery of conformational distributions through reweighting, while unreweighted results can be used to sample low-energy physical state provide a useful semi-quantitative ranking of their probabilities. For our purposes, here we analyze unreweighted results, as in our previous paper on the off-target effects in CRISPR-Cas9,[3] such obtaining a broad exploration of the conformational dynamics.

Based on extensive testing, performed in our previous study on the CRISPR-Cas9 conformational dynamics,[3,6,8] the system threshold energy has be set to $E = V_{max}$ for all Gaussian aMD simulations. The boost potential has been applied in a dual-boost scheme, in which two acceleration potentials are applied simultaneously to the system: (i) the torsional terms only and (ii) across the entire potential. A timestep of 2 fs has been used. Given an average system size of ~220K atoms, the maximum, minimum, average and standard deviation values of

the system potential (V_{max}, V_{min}, V_{avg} and σ_V) has been obtained from an initial ~100 ns NVT simulation with no boost potential (see details above). Each Gaussian aMD simulation proceeded with a ~50 ns run, in which the boost potential has been updated every 1.6 ns, thus reaching equilibrium values. Finally, ~1 μs of Gaussian aMD simulations have been carried out in the NVT ensemble for each system, in analogy to our previous paper,[3] to enable proper comparison.

Analysis of the RNA:DNA hybrid structure. Analysis of the RNA:DNA dynamics has been done over the Gaussian aMD production runs using the CURVES+ code.[28] As a measure of the base pair complementarity, we computed the *Propeller Twist* angle, which describes the rotation of couples of base pairs with respect to each other. Based on our previous study,[3] this parameter enables to properly characterize alterations in the base pairing along the RNA:DNA hybrid. The computed *Propeller Twist* angles have been plotted employing "violin plots", which provide an overall view of their summary statistics and probability distribution. The minor groove has been measured between cubic spline curves running through the phosphorus atoms of the nucleic backbone and then reduced by 5.8 Å (2 x 2.9 Å) to discount the average radius of two adjacent phosphodiester backbones. Analysis of the minor groove width includes the calculation of the statistical error at each level of the RNA:DNA hybrid (Figures 2 and S2). The analysis of the results has been performed over the last ~800 ns of GaMD, as in our previous paper,[3] which is used as a comparison for the current paper. This choice has been motivated by the analysis of the RMSD of the RNA:DNA hybrid, which stabilizes after the first ~200 ns of GaMD (Figure S1). Hence, the last converged part (i.e., last ~800 ns) of the simulated runs has been object of analysis. To father validate this choice, the conformational changes of the RNA:DNA hybrid structure have also been analyzed over the last ~400 ns of GaMD, reporting no significant difference with the analysis performed over the last ~800 ns (Figure S2).

Principal Component Analysis (PCA). In PCA, the covariance matrix of the protein Cα atoms is calculated and diagonalized to obtain a new set of generalized coordinates (eigenvectors) to describe the system motions. Each eigenvector – also called Principal Component (PC) – is associated to an eigenvalue corresponding to the mean square fluctuation contained in the system's trajectory projected along that eigenvector. By sorting the eigenvectors according to their eigenvalues, the first few Principal Components (PCs) corresponds to the system's largest amplitude motion (variance), and the dynamics of the system along these PCs is referred as *"essential dynamics"*.[29] Here, each conformation of the HNH domain sampled during the Gaussian aMD trajectories is projected into the collective coordinate space defined by the first two eigenvectors (PC1 and PC2), such allowing the characterization of the essential conformational sub-space sampled by Cas9 during Gaussian aMD. Importantly, each simulated system has been superposed onto the same reference structure and aligned, such allowing the projection into the same collective coordinate space. PCA has been performed using cpptraj of Amber18,[16] while the Normal Mode Wizard plugin of the Visual Molecular Dynamics[30] program has been used for the graphical rendering in Figure 3.

Cross-Correlation analysis. Cross-Correlation (CC_{ij}) analysis has been performed in order to identify the coupling of the motions between the residues of the HNH domain and of the DNA TS. The CC_{ij} coefficients have been computed between the Cα atoms of the HNH domain (i) and the TS phosphate atoms (j), as follows:

$$CC_{ij} = \frac{\langle \Delta\vec{r_i}(t) \cdot \Delta\vec{r_j}(t) \rangle}{\left(\langle \Delta\vec{r_i}(t)^2 \rangle \langle \Delta\vec{r_j}(t)^2 \rangle \right)^{\frac{1}{2}}} \quad [6]$$

where Δr_i and Δr_j are the fluctuation vectors of the atoms i and j, respectively. The angle brackets represent an average over the sampled time period. The value of CC_{ij} ranges from -1 to

1. Positive CC_{ij} values describe a correlated motion between atoms *i* and *j*, while negative CC_{ij} values describe anti-correlated motions. The CC_{ij} have been computed between the residues of the HNH domain that locate in proximity of the hybrid (i.e., residues 890-900, 901-910 and 911-920, which form three α-helices, Figure 4) and the TS bases from position b20 to b9, and have been plotted as a 2x2 matrix (Figure 4).

Supplementary Figures

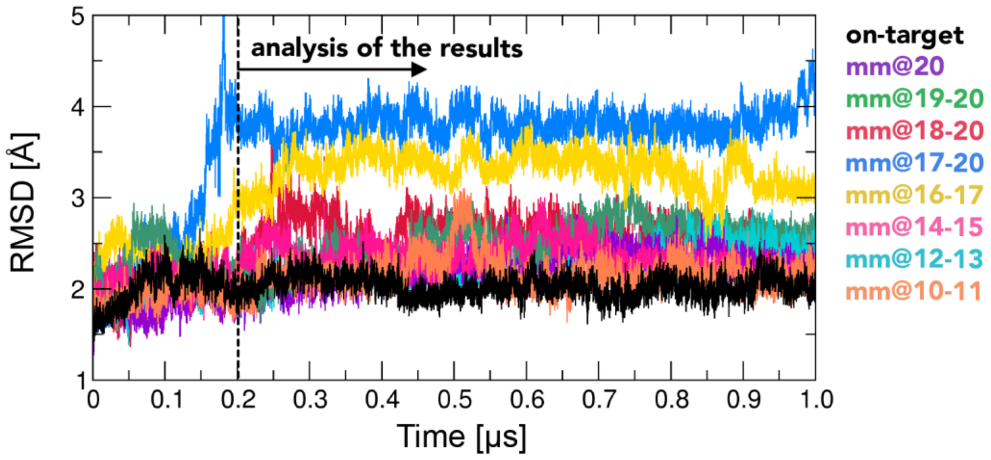

Figure S1. Time evolution of the Root Mean Square Deviation (RMSD) of the RNA:DNA hybrid structure, along Gaussian accelerated MD (GaMD) of the CRISPR-Cas9 system including the on-target DNA (i.e., on-target system) and base pair mismatches (mm) at different positions of the hybrid (i.e., mm@20, mm@19−20, mm@18−20, mm@17−20, mm@16−17, mm@14−15, mm@12−13 and mm@10−11 systems). The RMSD of the RNA:DNA hybrid stabilizes after the first ~0.2 μs of GaMD. Hence, the last converged ~0.8 μs have been considered for analysis. Notably, the mm@17−20 and mm@16−17 systems display increased RMSD values, due to the fact that the RNA:DNA hybrid undergoes structural changes.

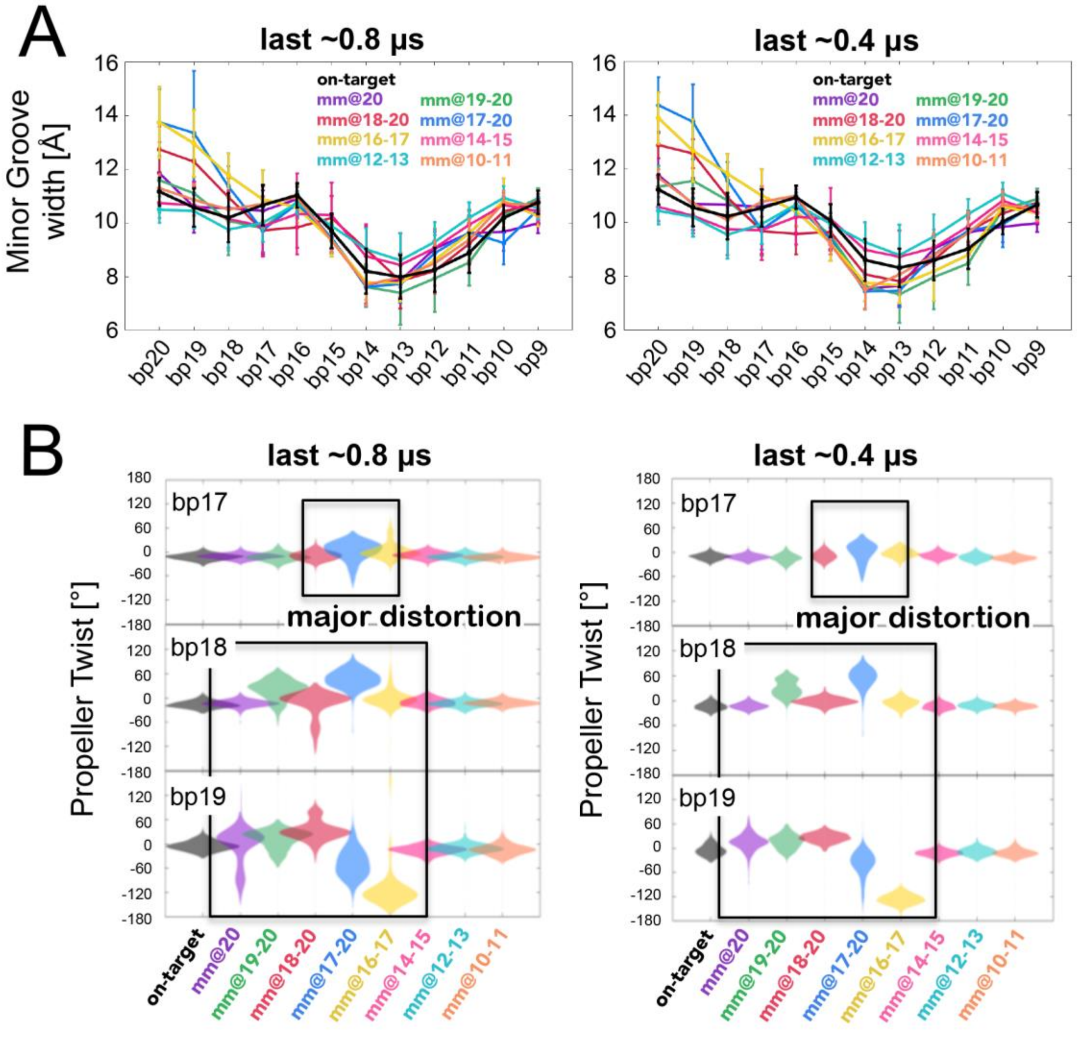

Figure S2. Analysis of the conformations adopted by the RNA:DNA hybrid over the last ~0.8 μs of GaMD (left panel) and over the last ~0.4 μs of GaMD (right panel). **(A)** Minor groove width measured at different levels of the RNA:DNA hybrid (i.e., from base pair 20 to 9) of the CRISPR-Cas9 system including the on-target DNA (i.e., on-target system) and base pair mismatches (mm) at different positions of the hybrid (i.e., mm@20, mm@19−20, mm@18−20, mm@17−20, mm@16−17, mm@14−15, mm@12−13 and mm@10−11 systems). **(B)** Probability distribution (as violin plot) of the *Propeller Twist* angle for the base pairs (bp) at positions 17 (top graph), 18 (central graph) and 19 (bottom graph) of the RNA:DNA hybrid.

Supplementary References

(1) Anders, C.; Niewoehner, O.; Duerst, A.; Jinek, M. Structural Basis of PAM-Dependent Target DNA Recognition by the Cas9 Endonuclease. *Nature* **2014**, *513* , 569–573.

(2) Dagdas, Y. S.; Chen, J. S.; Sternberg, S. H.; Doudna, J. A. A Conformational Checkpoint between DNA Binding and Cleavage by CRISPR-Cas9. *Sci. Adv.* **2017**, *3*, eaao002.

(3) Ricci, C. G.; Chen, J. S.; Miao, Y.; Jinek, M.; Doudna, J. A.; McCammon, J. A.; Palermo, G. Deciphering Off-Target Effects in CRISPR-Cas9 through Accelerated Molecular Dynamics. *ACS Cent. Sci.* **2019**, *5*, 651–662.

(4) Miao, Y.; Feher, V. A.; McCammon, J. A. Gaussian Accelerated Molecular Dynamics: Unconstrained Enhanced Sampling and Free Energy Calculation. *J. Chem. Theor. Comput.* **2015**, *11*, 3584–3595.

(5) Palermo, G.; Ricci, C. G.; Fernando, A.; Basak, R.; Jinek, M.; Rivalta, I.; Batista, V. S.; McCammon, J. A. Protospacer Adjacent Motif-Induced Allostery Activates CRISPR-Cas9. *J. Am. Chem. Soc.* **2017**, *139*, 16028–16031.

(6) Palermo, G.; Miao, Y.; Walker, R. C.; Jinek, M.; McCammon, J. A. CRISPR-Cas9 Conformational Activation as Elucidated from Enhanced Molecular Simulations. *Proc. Natl. Acad. Sci.* **2017**, *114* (28), 7260–7265. https://doi.org/10.1073/pnas.1707645114.

(7) Palermo, G.; Miao, Y.; Walker, R. C.; Jinek, M.; McCammon, J. A. Striking Plasticity of CRISPR-Cas9 and Key Role of Non-Target DNA, as Revealed by Molecular Simulations. *ACS Cent. Sci.* **2016**, *2*, 756–763.

(8) Palermo, G. Structure and Dynamics of the CRISPR–Cas9 Catalytic Complex. *J. Chem. Inf. Model.* **2019**, *59*, 2394–2406.

(9) Palermo, G.; Cavalli, A.; Klein, M. L.; Alfonso-Prieto, M.; Dal Peraro, M.; De Vivo, M.

Catalytic Metal Ions and Enzymatic Processing of DNA and RNA. *Acc. Chem. Res.* **2015**, *48*, 220–228.

(10) Perez, A.; Marchan, I.; Svozil, D.; Sponer, J.; Cheatham, T. E. 3rd; Laughton, C. A.; Orozco, M. Refinement of the AMBER Force Field for Nucleic Acids: Improving the Description of Alpha/Gamma Conformers. *Biophys. J.* **2007**, *92*, 3817–3829.

(11) Banas, P.; Hollas, D.; Zgarbova, M.; Jurecka, P.; Orozco, M.; Cheatham, T. E. 3rd; Sponer, J.; Otyepka, M. Performance of Molecular Mechanics Force Fields for RNA Simulations: Stability of UUCG and GNRA Hairpins. *J. Chem. Theor. Comput.* **2010**, *6*, 3836–3849.

(12) Zgarbova, M.; Otyepka, M.; Sponer, J.; Mladek, A.; Banas, P.; Cheatham, T. E.; Jurecka, P. Refinement of the Cornell et Al. Nucleic Acids Force Field Based on Reference Quantum Chemical Calculations of Glycosidic Torsion Profiles. *J. Chem. Theory Comput.* **2011**, *7*, 2886–2902.

(13) Aqvist, J. Ion-Water Interaction Potentials Derived from Free Energy Perturbation Simulations. *J. Phys. Chem.* **1990**, *94*, 8021–8024.

(14) Turq, P.; Lantelme, F.; Friedman, H. L. Brownian Dynamics: Its Applications to Ionic Solutions. *J. Chem. Phys.* **1977**, *66*, 3039.

(15) Berendsen, H. J. C.; Postma, J. P. M.; van Gunsteren, W. F.; DiNola, A.; Haak, J. R. Molecular Dynamics with Coupling to an External Bath. *J. Chem. Phys.* **1984**, *81*, 3684.

(16) Case, D. A.; Betz, R. M.; Botello-Smith, W.; Cerutti, D. S.; Cheatham T. E., I. I. I.; Darden, T. A.; Duke, R. E.; Giese, T. J.; Gohlke, H.; Goetz, A. W.; et al. AMBER 2016. *Univ. California, San Fr.* **2016**.

(17) Hamelberg, D.; Mongan, J.; McCammon, J. A. Accelerated Molecular Dynamics: A

Promising and Efficient Simulation Method for Biomolecules. *J. Chem. Phys.* **2004**, *120*, 11919–11929.

(18) Wereszczynski, J.; Mccammon, J. A. Nucleotide-Dependent Mechanism of Get3 as Elucidated from Free Energy Calculations. **2012**. *Proc. Natl. Acad. Sci. U. S. A.* **2012**, *15*, 7759–7764.

(19) Fajer, M.; Hamelberg, D.; McCammon, J. A. Replica-Exchange Accelerated Molecular Dynamics (REXAMD) Applied to Thermodynamic Integration. *J. Chem. Theory Comput.* **2008**, *4*, 1565–1569.

(20) Jiang, W.; Thirman, J.; Jo, S.; Roux, B. Reduced Free Energy Perturbation/Hamiltonian Replica Exchange Molecular Dynamics Method with Unbiased Alchemical Thermodynamic Axis. *J. Phys. Chem. B* **2018**, *122*, 9435–9442.

(21) Miao, Y.; Sinko, W.; Pierce, L.; Bucher, D.; Walker, R. C.; McCammon, J. A. Improved Reweighting of Accelerated Molecular Dynamics Simulations for Free Energy Calculation. *J. Chem. Theory Comput.* **2014**, *10*, 2677–2689.

(22) Miao, Y.; Feixas, F.; Eun, C.; McCammon, J. A. Accelerated Molecular Dynamics Simulations of Protein Folding. *J. Comput. Chem.* **2015**, *36*, 1536–1549.

(23) Miao, Y.; McCammon, J. A. Mechanism of the G-Protein Mimetic Nanobody Binding to a Muscarinic G-Protein-Coupled Receptor. *Proc. Natl. Acad. Sci. USA* **2018**, *115*, 3036–3041.

(24) Miao, Y.; McCammon, J. A. Graded Activation and Free Energy Landscapes of a Muscarinic G Protein-Coupled Receptor. *Proc Natl Acad Sci U S A* **2016**, *113*, 12162–12167.

(25) Liao, J. M.; Wang, Y. T. In Silico Studies of Conformational Dynamics of Mu Opioid

Receptor Performed Using Gaussian Accelerated Molecular Dynamics. *J Biomol Struct Dyn* **2018**, 1–12.

(26) Wang, Y.-T.; Chan, Y.-H. Understanding the Molecular Basis of Agonist/Antagonist Mechanism of Human Mu Opioid Receptor through Gaussian Accelerated Molecular Dynamics Method. *Sci. Rep.* **2017**, *7*, 7828.

(27) Sibener, L. V.; Fernandes, R. A.; Kolawole, E. M.; Carbone, C. B.; Liu, F.; McAffee, D.; Birnbaum, M. E.; Yang, X.; Su, L. F.; Yu, W.; et al. Isolation of a Structural Mechanism for Uncoupling T Cell Receptor Signaling from Peptide-MHC Binding. *Cell* **2018**, *174*, 672-687.

(28) Lavery, R.; Moakher, M.; Maddocks, J. H.; Petkeviciute, D.; Zakrzewska, K. Conformational Analysis of Nucleic Acids Revisited: Curves+. *Nucleic Acids Res.* **2009**, *37*, 5917–5929.

(29) Amadei, A.; Linssen, A. B. M.; Berendsen, H. J. C. Essential Dynamics of Proteins. *Proteins Struct. Funct. Genet.* **1993**, *17*, 412–425.

(30) Humphrey, W.; Dalke, A.; Schulten, K. VMD: Visual Molecular Dynamics. *J Mol Graph* **1996**, *14*, 27-28.
